# The complete mitochondrial genome of *Schizothorax eurystomus* Kessler, 1872 (Cypriniformes, Cyprinidae)

**DOI:** 10.1080/23802359.2022.2142071

**Published:** 2022-11-11

**Authors:** Jiangong Niu, Hong Liu, Jiangwei Hu, Tao Zhang, Hui Zhang

**Affiliations:** a Xinjiang Fisheries Research Institute, Urumqi, PR China; b Ministry of Agriculture, Scientific Observing and Experimental Station of Fishery Resources and Environment in Northwest China, Urumqi, PR China; c CAS Key Laboratory of Marine Ecology and Environmental Sciences, Institute of Oceanology, Chinese Academy of Sciences, Qingdao, PR China

**Keywords:** *Schizothorax eurystomus*, mitochondrial genome, NGS, freshwater fish

## Abstract

*Schizothorax eurystomus*, Kessler 1872 is a unique economic fish in Xinjiang, China that is rarely seen in the market. Next-generation sequencing (NGS) was used to determine the complete mitochondrial genome of *S. eurystomus* collected from the Yarkand River in Xinjiang. The results showed that the mitochondrial genome is a circular, 16,488-bp-long nucleotide with the typical vertebrate genome structure of 13 protein-coding genes, 2 ribosomal RNA genes, 22 transfer RNA genes, and a control region. The termination-associated sequence (TAS), central conserved sequence block (CSB), and conserved sequence block were detected in the control region. Phylogenetic analysis placed *S. eurystomus* in a fully supported clade with *S. biddulphi*, and that clade was sister to *S. yunnanensis.* To our knowledge, this is the first study on the complete mitochondrial genome of *S. eurystomus* from the Yarkand River in Xinjiang, and it provides baseline genetic information for future studies.

*Schizothorax eurystomus*, Kessler 1872, also known as the wide-mouth hip scale fish, is a cold-water white fish that belongs to order Cypriniformes, family Cyprinidae, and subfamily Schizothoracinae (Cao et al. 2019). The *S. eurystomus* body is lengthened and slightly laterally flattened, and its head is tapered; *S. eurystomus* characteristics are shown in Figure 1. *S. eurystomus* used to be the main economic fish in the Tarim River and its tributaries and affiliated lakes (Cao et al. 2019). Recently, with the impact of hydropower development and human activities, the *S. eurystomus* resources are decreasing, and the Xinjiang autonomous region plans to list it as a protected fish (Nie et al. 2014).

**Figure 1.**
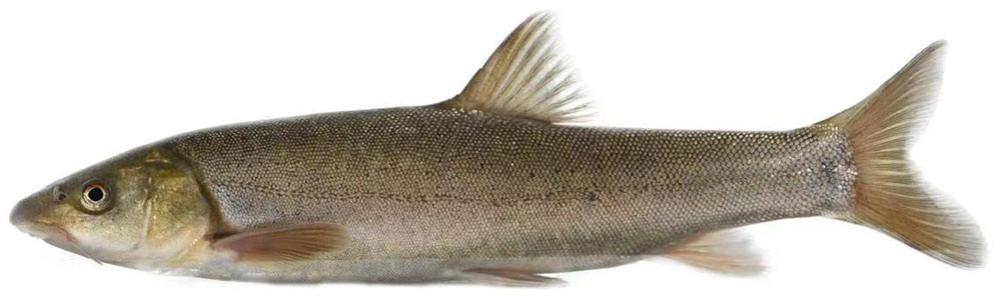
*Schizothorax eurystomus*.

Next-generation sequencing (NGS) has revolutionized the field of molecular biology because it is rapid and can generate large amounts of genomic data (Schuster 2008; Koboldt et al. 2013). Therefore, in this study, the complete mitochondrial genome of *S. eurystomus* was determined using NGS technology to provide insight into the population processes and evolutionary history of this species (Zhang and Xian 2015).

DNA was extracted from muscle tissue of *S. eurystomus* collected from Altash Station (76.29E, 37.59 N) of the Xinjiang Yarkand River in September 2021. A specimen was deposited at the CAS Key Laboratory of Marine Ecology and Environmental Sciences, Institute of Oceanology, Chinese Academy of Sciences (Hui Jia, jiahui@qdio.ac.cn) under voucher number L212. The Illumina NovaSeq sequencing platform (Illumina, San Diego, CA) was used to process sequences.

The high-quality second-generation genome sequence was assembled and analyzed using A5-miseq v20150522 (Coil et al. 2015) and SPAdes version 3.9.0 (Bankevich et al. 2012). Pilon version 1.18 (Walker et al. 2014) was used to correct the results and obtain the final mitochondrial sequence. Annotation of the complete mitochondrial genome sequence was performed on the MITOS web server (http://mitos2.bioinf.uni-leipzig.de/index.py) (Bernt et al. 2013). After sequencing, the maximum likelihood (ML) method with K2P distances was used to construct the ML tree in MEGA10 with 1000 bootstrap replicates (Kumar et al. 2018).

Similar to the size of other teleost mitogenomes, the complete mitochondrial genome of *S. eurystomus* was 16,488-bp long (GenBank accession ON920824). The circular map of the complete *S. eurystomus* mitochondrial genome is shown in Figure 1. The complete mitochondrial genome contained 13 protein-coding genes, 2 ribosomal RNA (rRNA) genes (12S rRNA and 16S rRNA), 22 transfer RNA (tRNA) genes, and a control region, similar to other vertebrates (Miya et al. 2001).

Among the 13 protein-coding genes, ATP6 and ATP8 overlapped by 7 nucleotides, and NAD4 and NAD4L shared 7 nucleotides. The NAD5 and NAD6 genes overlapped by 4 nucleotides on the opposite strand. Most of the genes of *S. eurystomus* were encoded on the heavy-strand (H-strand), with only NAD6 and 8 tRNA genes (*tRNA^Gln^*, *tRNA^Ala^*, *tRNA^Asn^*, *tRNA^Cys^*, *tRNA^Tyr^*, *tRNA^Ser^*, *tRNA^Glu^*, and *tRNA^Pro^*) encoded on the light-strand (L-strand) (Table 1). The ATG codon initiated 12 of the 13 protein-coding genes (*NAD1*, *NAD2*, *COII*, *ATP8*, *ATP6*, *COIII*, *NAD3*, *NAD4L*, *NAD4*, *NAD5*, *NAD6*, and *COB*), and GTG was the initiation codon for COI (Figure 2). The stop codon TAA terminated seven genes (*NAD1*, *COI*, *AYP6*, *COIII*, *NAD4L*, *NAD5*, and *NAD6*), TAG terminated three genes (*NAD2*, *ATP8*, and *NAD3*); the incomplete T– terminated the *COII*, *NAD4*, and *COB* genes by post-transcriptional polyadenylation (Ojala et al. 1981).

**Table 1.** Analysis of mitochondrial geneome characteristics.

Feature	Strand	Position	Length (bp)	Initiation codon	Stop codon	Anticodon	Intergenic nucleotide
trnF	N	1–69	69			GAA	
rrnS	N	70–1025	956				2
trnV	N	1028–1099	72			TAC	22
rrnL	N	1122–2751	1630				24
trnL2	N	2776–2851	76			TAA	
nad1	N	2852–3826	975	ATG	TAA		4
trnI	N	3831–3902	72			GAT	−2
trnQ	J	3901–3971	71			TTG	2
trnM	N	3974–4042	69			CAT	
nad2	N	4043–5089	1047	ATG	TAG		−2
trnW	N	5088–5158	71			TCA	2
trnA	J	5161–5229	69			TGC	1
trnN	J	5231–5303	73			GTT	2
OL	N	5306-5,337	32				−1
trnC	J	5337–5403	67			GCA	−1
trnY	J	5403–5473	71			GTA	1
cox1	N	5475–7025	1551	GTG	TAA		
trnS2	J	7026–7096	71			TGA	3
trnD	N	7100–7171	72			GTC	13
cox2	N	7185–7875	691	ATG	T(AA)		
trnK	N	7876–7951	76			TTT	1
atp8	N	7953–8117	165	ATG	TAG		−7
atp6	N	8111–8794	684	ATG	TAA		−1
cox3	N	8794–9579	786	ATG	TAA		−1
trnG	N	9579–9650	72			TCC	
nad3	N	9651–10,001	351	ATG	TAG		−2
trnR	N	10,000–10,069	70			TCG	
nad4l	N	10,070–10,366	297	ATG	TAA		−7
nad4	N	10,360–11,740	1381	ATG	T(AA)		
trnH	N	11,741–11,809	69			GTG	
trnS1	N	11,810–11,877	68			GCT	1
trnL1	N	11,879–11,951	73			TAG	3
nad5	N	11,955–13,778	1824	ATG	TAA		−4
nad6	J	13,775–14,296	522	ATG	TAA		
trnE	J	14,297–14,365	69			TTC	4
cob	N	14,370–15,510	1141	ATG	T(AA)		
trnT	N	15,511–15,582	72			TGT	−1
trnP	J	15,582–15,651	70			TGG	16
OH	N	15,668–16,488	821				101

The 12S and 16S rRNA genes of *S. eurystomus* were 956- and 1630-bp long, respectively. They were located between tRNA^Phe^ and tRNA^Leu^, and were separated by tRNA^Val^, similar to other vertebrates (Zhang et al. 2016). The 22 tRNA genes were scattered throughout the genome, ranging 67–76 bp and folded into a cloverleaf secondary structure with normal base shedding. The major non-coding region in *S. eurystomus* was located between tRNA^Pro^ and tRNA^Phe^, and was 821-bp long. The termination-associated sequence (TAS), central conserved sequence block (CSB), and CSB were detected in the control region and were similar to those of most bony fishes (Jia et al. 2021).

One complete mitogenome of *S. eurystomus* has been deposited in GenBank (https://www.ncbi.nlm.nih.gov/nuccore/KY436758.1), however, there is no paper published for the description in details on it. In this study, we collected and identified five specimens, and then used NGS to obtain their mitochondrial genome. The complete sequences were used for phylogenetic analysis and the phylogenetic results revealed that a previously collected specimen (KY436758.1) was genetically distant from other related species; therefore, the complete mitochondrial genome of *S. eurystomus* needed to be updated. Phylogenetic analysis of the complete mitochondrial genome of *S. eurystomus* revealed that it belonged to a clade with *S. biddulphi*, and they are sister to *S. yunnanensis* (Figure 3). This study is the first to phylogenetically analyze *S. eurystomus* in detail and provides a scientific basis for future molecular systematic and phylogenetic studies of bony fishes in Cyprinidae.[Fig F0003]

**Figure 2.**
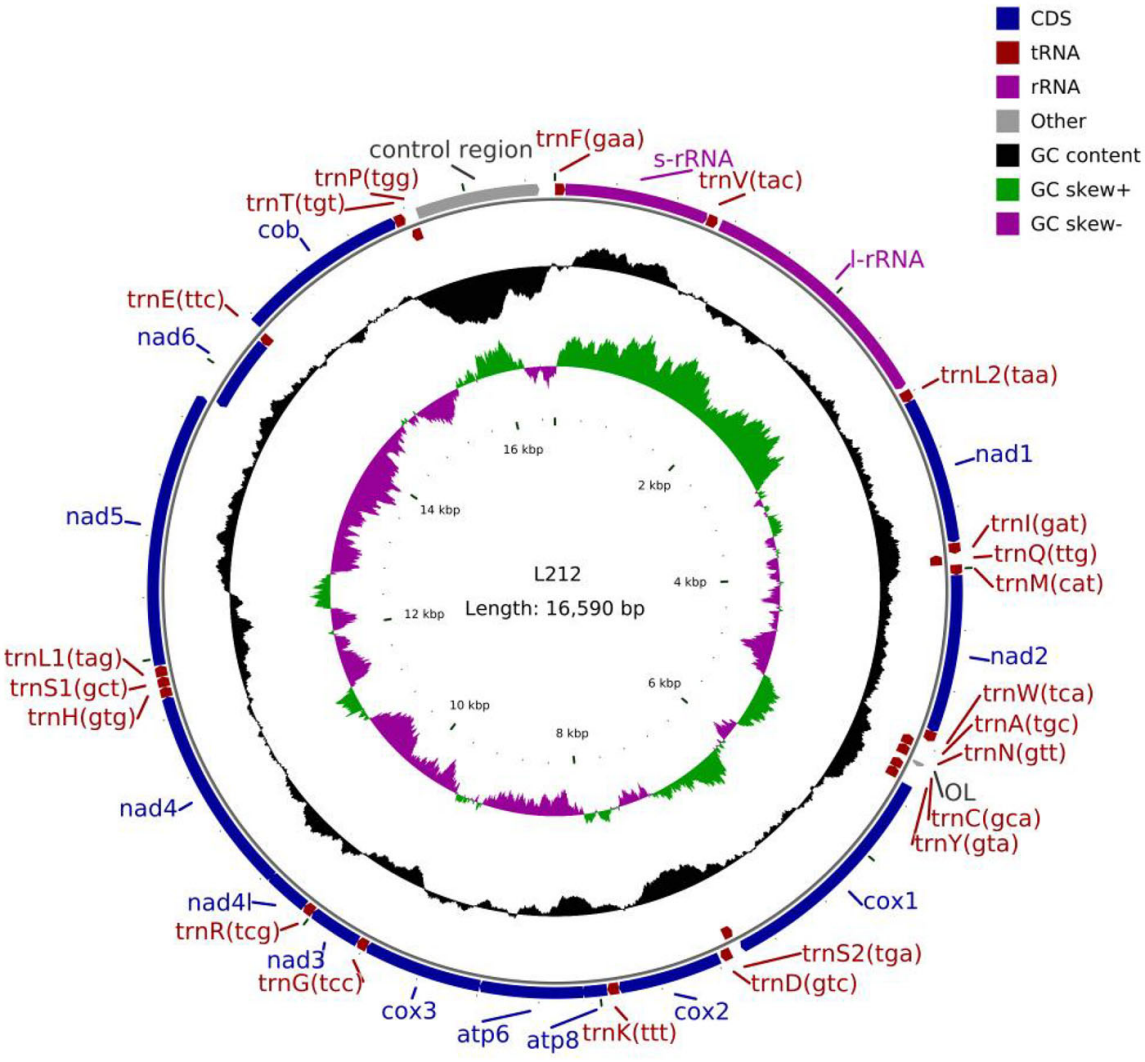
Complete mitochondrial genome circle map of *Schizothorax eurystomus.*

**Figure 3. F0003:**
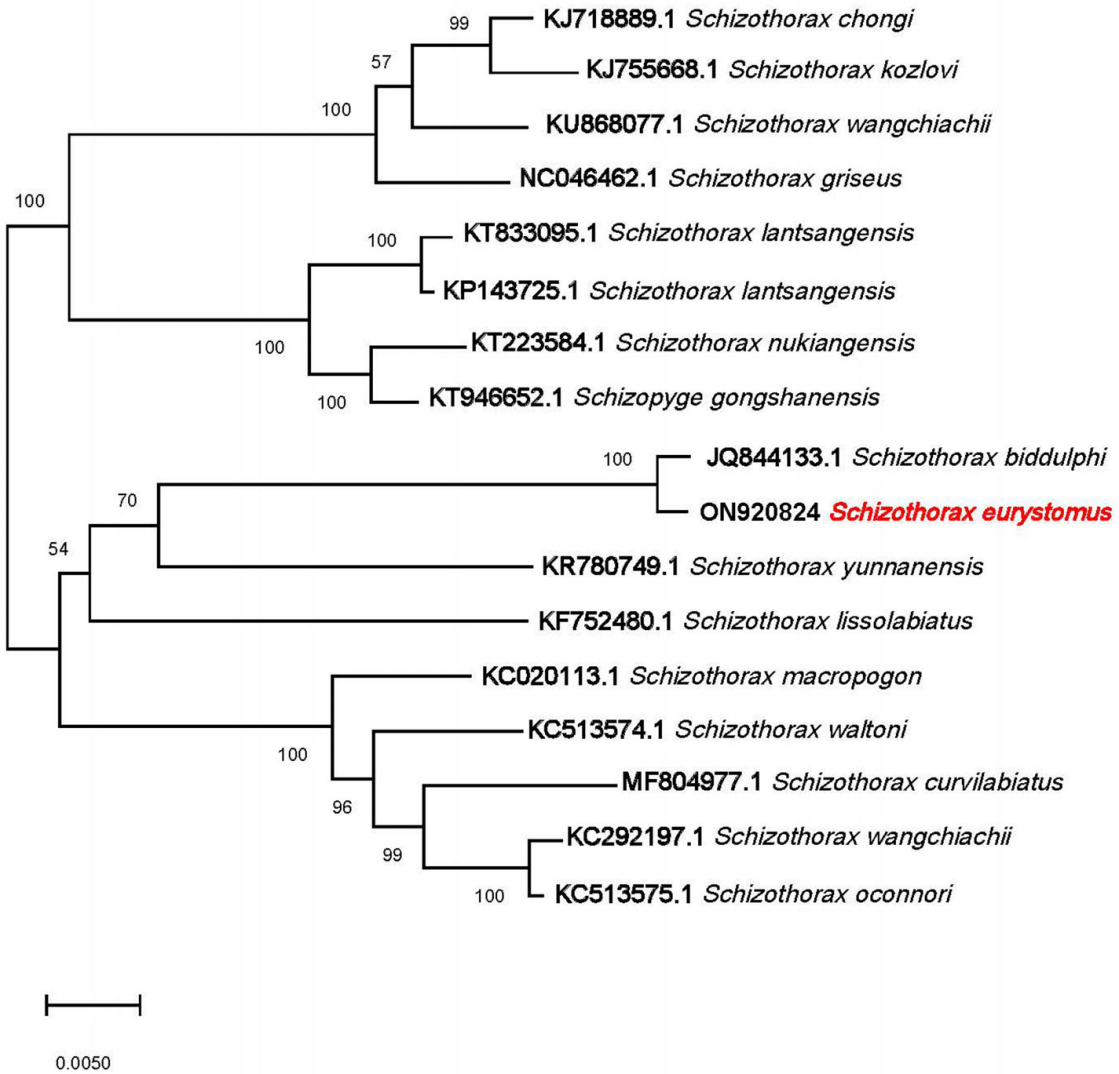
Maximum likelihood tree of *Schizothorax eurystomus* and seventeen related Cypriniformes based on complete mitogenomes. Bootstrap values based on 1000 replicates and are represented at the nodes.

## Data Availability

Mitogenome data supporting this study are openly available in GenBank at nucleotide database, https://www.ncbi.nlm.nih.gov/nuccore/ON920824, Associated BioProject, https://www.ncbi.nlm.nih.gov/bioproject/PRJNA855635, BioSample accession number at https://www.ncbi.nlm.nih.gov/biosample/ SAMN29499325 and Sequence Read Archive at https://www.ncbi.nlm.nih.gov/sra/SRR19976742.
